# Epidemics—Circular

**Published:** 1853-09

**Authors:** 


EPIDEMICS—CIRCULAR.

Dr. W. L. Sutton, Chairman of the Committee on the Epidemics of Kentucky and Tennessee, respectfully tenders his thanks to those physicians of the two States who were so kind as to furnish materials towards making out the report for 1851 and 1852, to-wit:

Dr. F. A. Ramsey, Knoxville, Dr. Lewis Shanks, Memphis, Dr. B. B. Lenoir, Roane Co., Dr. Geo. R Grant, Memphis, Dr. E. B. Haskins, Clarksville, Dr. J. V. W. Conn, Pelham, Dr. W. W. McNelly, Fayettville, Dr. Thomas Lipscomb, Shelbyville, Tennessee; and Dr. Jno. Swain, Ballardsville, Dr. B. F. Stevenson, Burlington, Dr. A. W. Newton, Boone Co., Dr. Jno. Sutton, Dr. H. C. Catlett, Dr. ——— Alexander, Hickman Co., Drs. Evans and Chambers, Covington, Dr. S. G. Ray and Son, Paris, Dr. Joseph Smith, Danville, Dr. W. R. Chew, Midway, Dr. J. V. Withers, Brandenburg, Dr. S. T. Newman, Richmond, Dr. A. Addams, Cynthiana, Dr. N. G. Dille, Harrrison Co., Dr. Jos. H. Hawkins, Colemansville, Dr. G. Hurt, Prestonsburg, Dr. James Armstrong, Liberty, and Dr. J. L. Ford, Brownsville, Kentucky.

It is earnestly hoped that all these gentlemen will continue their favors, and that others, *many others,* will imitate their praiseworthy example and furnish the result of their observation, upon such epidemics as may prevail during the next *four years,* in their respective neighborhoods

Dr. Sutton would respectfully state that all communications will be thankfully recieved at any time; but if they could be furnished by the first of April, the obligation would be greatly enhanced. Last spring, several very valuable papers came to hand after the report was thought to be finished, and one after it had been sent on to the Medical Association. *Verbum sat.*

Georgetown Ky. Aug. 5th 1853.

